# 5-(Methoxy­carbon­yl)thio­phene-2-carboxylic acid

**DOI:** 10.1107/S1600536809053161

**Published:** 2009-12-16

**Authors:** Guang-Ming Xia, Mu-Wei Ji, Ping Lu, Guo-Xin Sun, Wen-Fang Xu

**Affiliations:** a School of Chemistry and Chemical Engineering, University of Jinan, Ji’nan 250022, People’s Republic of China; b School of Pharmaceutical Sciences, Shandong University, Ji’nan 250012, People’s Republic of China

## Abstract

In the title compound, C_7_H_6_O_4_S, a monoester derivative of 2,5-thio­phene­dicarboxylic acid, the carboxylic acid and the carboxylic acid ester groups are approximately coplanar with thio­phene ring, making a dihedral angle of 3.1 (4) and 3.6 (4)°, respectively. In the crystal structure, mol­ecules are connected by classical inter­molecular O—H⋯O hydrogen bonds, forming centrosymmetric dimers.

## Related literature

For a related structure, see: Zhao *et al.* (2009 ▶).
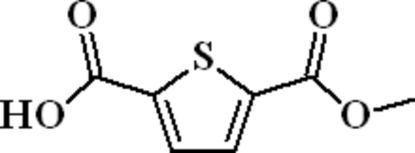


## Experimental

### 

#### Crystal data

C_7_H_6_O_4_S
*M* *_r_* = 186.19
Monoclinic, 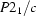

*a* = 18.2813 (18) Å
*b* = 5.9833 (6) Å
*c* = 7.3446 (8) Å
β = 99.081 (1)°
*V* = 793.30 (14) Å^3^
*Z* = 4
Mo *K*α radiation
μ = 0.38 mm^−1^
*T* = 298 K
0.40 × 0.28 × 0.12 mm

#### Data collection

Siemens SMART APEX CCD area-detector diffractometer
Absorption correction: multi-scan (*SADABS*; Sheldrick, 1996 ▶) *T*_min_ = 0.864, *T*_max_ = 0.956
3914 measured reflections
1398 independent reflections
958 reflections with *I* > 2σ(*I*)
*R*_int_ = 0.035

#### Refinement

*R*[*F* ^2^ > 2σ(*F* ^2^)] = 0.041
*wR*(*F* ^2^) = 0.108
*S* = 0.96
1398 reflections
110 parameters
H-atom parameters constrained
Δρ_max_ = 0.19 e Å^−3^
Δρ_min_ = −0.26 e Å^−3^

### 

Data collection: *SMART* (Siemens, 1996 ▶); cell refinement: *SAINT* (Siemens, 1996 ▶); data reduction: *SAINT*; program(s) used to solve structure: *SHELXS97* (Sheldrick, 2008 ▶); program(s) used to refine structure: *SHELXL97* (Sheldrick, 2008 ▶); molecular graphics: *SHELXTL* (Sheldrick, 2008 ▶); software used to prepare material for publication: *SHELXTL*.

## Supplementary Material

Crystal structure: contains datablocks global, I. DOI: 10.1107/S1600536809053161/rk2181sup1.cif

Structure factors: contains datablocks I. DOI: 10.1107/S1600536809053161/rk2181Isup2.hkl

Additional supplementary materials:  crystallographic information; 3D view; checkCIF report

## Figures and Tables

**Table 1:** Hydrogen-bond geometry (Å, °)

*D*—H⋯*A*	*D*—H	H⋯*A*	*D*⋯*A*	*D*—H⋯*A*
O4—H4⋯O3^i^	0.82	1.82	2.639 (2)	173

Symmetry code: (i) 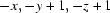.

**Fig. 1.**
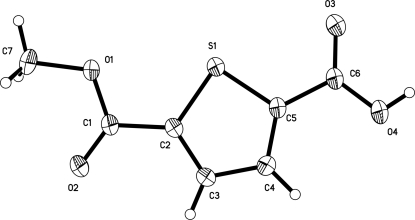
Molecular structure of title compound with the atom numbering scheme. Displacement ellipsoids are drawn at 30% probability level. H atoms are presented as a small spheres of arbitrary radius.

**Fig. 2.**
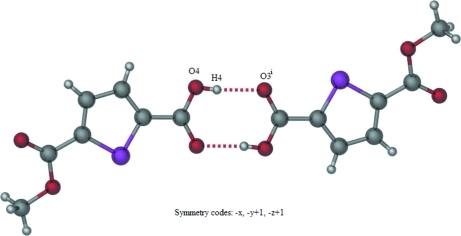
The centrosymmetrical H–bonded dimer. Hydrogen bonds presented by dashed lines. Symmetry code: (i) -x, -y+1, -z+1.

## supplementary crystallographic information

### Comment

The derivates of thiophene have been viewed as significant compounds for application in many fields, such as photo–material, electronic luminescence material (Zhao *et al.*, (2009)). Many simple structures containing thiophene ring were synthesized for their derivates. When substituted with different active function groups, a series of valuable derivates of thiophene can be obtained. It may be used as a source to synthesize compounds which has more complex structures. The title compound was synthesized as a promising compound with biological activities and a precursor for the synthesis of various functional compounds for its delocalized structure.

In the structure of the title compound (Fig. 1), the carboxylate groups are approximately coplanar with thiophene ring. The co–plane connection makes the π–conjugation expanded in a larger range. In the crystal structure, molecules are connected by intermolecular O4—H4···O3^i^ hydrogen–bonding interactions (Table 1) forming a dimer (Fig. 2). Symmetry code: (i) -*x*, -*y*+1, -*z*+1.

### Experimental

Sodium (230 mg, 10 mmol) was dissolved in 40 ml of absolute methanol, the resulting solution was adopted into a solution of dimethylthiophen–2,5–dicarboxylate (2000 mg, 10 mmol) in 60 ml of absolute methanol. The resulting mixture was heated at 343 K for 5 h, cooled, and the filtrated. The filtrate was acidified with HCl (6 mol.*L*^-1^) to pH about 5. As the HCl being adopted, the product was formed as colourless solid (yield: 152 mg, 82%). Recrystallized with methanol at room temperature afforded colourless crystal. IR–spectrum (KBr): *v* = 3097, 1728, 1712 cm^-1^.

### Refinement

All H atoms were geometrically fixed and allowed to ride on their attached atoms, which C—H = 0.93–0.96Å and *U*_iso_(H) = 1.2–1.5*U*_eq_(C) and O—H = 0.82Å and *U*_iso_(H) = 1.2*U*_eq_(O).

### Crystal data

**Table:**

C_7_H_6_O_4_S	*F*(000) = 384
*M**_r_* = 186.19	*D*_x_ = 1.559 Mg m^−^^3^
Monoclinic, *P*2_1_/*c*	Mo *K*α radiation, λ = 0.71073 Å
Hall symbol: -P 2ybc	Cell parameters from 978 reflections
*a* = 18.2813 (18) Å	θ = 2.3–25.7°
*b* = 5.9833 (6) Å	µ = 0.38 mm^−^^1^
*c* = 7.3446 (8) Å	*T* = 298 K
β = 99.081 (1)°	Block, colourless
*V* = 793.30 (14) Å^3^	0.40 × 0.28 × 0.12 mm
*Z* = 4	

### Data collection

**Table:**

Siemens SMART APEX CCD area-detector diffractometer	1398 independent reflections
Radiation source: fine–focus sealed tube	958 reflections with *I* > 2σ(*I*)
graphite	*R*_int_ = 0.035
φ and ω scans	θ_max_ = 25.0°, θ_min_ = 2.3°
Absorption correction: multi-scan (*SADABS*; Sheldrick, 1996)	*h* = −17→21
*T*_min_ = 0.864, *T*_max_ = 0.956	*k* = −6→7
3914 measured reflections	*l* = −8→8

### Refinement

**Table:**

Refinement on *F*^2^	Primary atom site location: structure-invariant direct methods
Least-squares matrix: full	Secondary atom site location: difference Fourier map
*R*[*F*^2^ > 2σ(*F*^2^)] = 0.041	Hydrogen site location: inferred from neighbouring sites
*wR*(*F*^2^) = 0.108	H-atom parameters constrained
*S* = 0.96	*w* = 1/[σ^2^(*F*_o_^2^) + (0.0567*P*)^2^] where *P* = (*F*_o_^2^ + 2*F*_c_^2^)/3
1398 reflections	(Δ/σ)_max_ < 0.001
110 parameters	Δρ_max_ = 0.19 e Å^−^^3^
0 restraints	Δρ_min_ = −0.26 e Å^−^^3^

### Special details

**Table:**

Geometry. All s.u.'s (except the s.u. in the dihedral angle between two l.s. planes) are estimated using the full covariance matrix. The cell s.u.'s are taken into account individually in the estimation of s.u.'s in distances, angles and torsion angles; correlations between s.u.'s in cell parameters are only used when they are defined by crystal symmetry. An approximate (isotropic) treatment of cell s.u.'s is used for estimating s.u.'s involving l.s. planes.
Refinement. Refinement of *F*^2^ against ALL reflections. The weighted *R*–factor *wR* and goodness of fit *S* are based on *F*^2^, conventional *R*–factors *R* are based on *F*, with *F* set to zero for negative *F*^2^. The threshold expression of *F*^2^ > σ(*F*^2^) is used only for calculating *R*–factors(gt) *etc*. and is not relevant to the choice of reflections for refinement. *R*–factors based on *F*^2^ are statistically about twice as large as those based on *F*, and *R*–factors based on ALL data will be even larger.

### Fractional atomic coordinates and isotropic or equivalent isotropic displacement parameters (Å^2^)

**Table:**

	*x*	*y*	*z*	*U*_iso_*/*U*_eq_	
O1	0.36617 (8)	0.4062 (3)	1.0635 (2)	0.0477 (5)	
O2	0.40524 (9)	0.7617 (3)	1.0853 (3)	0.0579 (5)	
O3	0.07899 (9)	0.3919 (3)	0.6150 (2)	0.0593 (6)	
O4	0.04479 (9)	0.7517 (3)	0.5849 (3)	0.0609 (6)	
H4	0.0068	0.6974	0.5278	0.091*	
S1	0.22488 (3)	0.47279 (11)	0.83803 (9)	0.0437 (3)	
C1	0.35915 (12)	0.6246 (4)	1.0321 (3)	0.0398 (6)	
C2	0.28593 (12)	0.6776 (4)	0.9231 (3)	0.0381 (6)	
C3	0.25867 (13)	0.8855 (4)	0.8830 (3)	0.0419 (6)	
H3	0.2847	1.0162	0.9174	0.050*	
C4	0.18615 (13)	0.8808 (4)	0.7830 (3)	0.0421 (7)	
H4A	0.1586	1.0079	0.7455	0.050*	
C5	0.16150 (12)	0.6693 (4)	0.7481 (3)	0.0400 (6)	
C6	0.09006 (13)	0.5956 (4)	0.6426 (3)	0.0432 (6)	
C7	0.43390 (14)	0.3361 (5)	1.1788 (4)	0.0597 (8)	
H7A	0.4753	0.3679	1.1174	0.090*	
H7B	0.4318	0.1784	1.2017	0.090*	
H7C	0.4395	0.4154	1.2938	0.090*	

### Atomic displacement parameters (Å^2^)

**Table:**

	*U*^11^	*U*^22^	*U*^33^	*U*^12^	*U*^13^	*U*^23^
O1	0.0345 (9)	0.0390 (11)	0.0619 (11)	0.0014 (8)	−0.0158 (8)	0.0037 (9)
O2	0.0401 (11)	0.0461 (12)	0.0783 (13)	−0.0098 (9)	−0.0190 (9)	0.0037 (10)
O3	0.0426 (11)	0.0456 (12)	0.0809 (14)	−0.0054 (9)	−0.0171 (9)	−0.0026 (10)
O4	0.0361 (10)	0.0523 (13)	0.0839 (13)	0.0006 (9)	−0.0226 (9)	−0.0035 (10)
S1	0.0357 (4)	0.0333 (4)	0.0563 (4)	−0.0016 (3)	−0.0105 (3)	−0.0007 (3)
C1	0.0310 (13)	0.0406 (16)	0.0446 (14)	−0.0017 (12)	−0.0045 (11)	0.0013 (12)
C2	0.0326 (13)	0.0355 (14)	0.0433 (14)	−0.0035 (10)	−0.0029 (11)	−0.0009 (11)
C3	0.0380 (14)	0.0327 (15)	0.0511 (15)	−0.0046 (12)	−0.0048 (11)	−0.0020 (12)
C4	0.0380 (14)	0.0349 (15)	0.0500 (15)	0.0040 (12)	−0.0034 (12)	0.0000 (12)
C5	0.0312 (14)	0.0393 (15)	0.0455 (14)	0.0007 (11)	−0.0063 (11)	0.0000 (12)
C6	0.0314 (13)	0.0434 (16)	0.0503 (15)	−0.0001 (13)	−0.0074 (11)	−0.0016 (13)
C7	0.0444 (16)	0.057 (2)	0.0694 (18)	0.0104 (14)	−0.0173 (13)	0.0124 (15)

### Geometric parameters (Å, °)

**Table:**

O1—C1	1.330 (3)	C2—C3	1.355 (3)
O1—C7	1.447 (3)	C3—C4	1.411 (3)
O2—C1	1.196 (3)	C3—H3	0.9300
O3—C6	1.247 (3)	C4—C5	1.354 (3)
O4—C6	1.275 (3)	C4—H4A	0.9300
O4—H4	0.8200	C5—C6	1.477 (3)
S1—C2	1.708 (2)	C7—H7A	0.9600
S1—C5	1.709 (2)	C7—H7B	0.9600
C1—C2	1.482 (3)	C7—H7C	0.9600
			
C1—O1—C7	115.88 (19)	C3—C4—H4A	124.0
C6—O4—H4	109.5	C4—C5—C6	128.2 (2)
C2—S1—C5	90.67 (12)	C4—C5—S1	112.63 (16)
O2—C1—O1	125.0 (2)	C6—C5—S1	119.13 (18)
O2—C1—C2	124.0 (2)	O3—C6—O4	125.6 (2)
O1—C1—C2	111.00 (19)	O3—C6—C5	119.0 (2)
C3—C2—C1	125.7 (2)	O4—C6—C5	115.4 (2)
C3—C2—S1	112.49 (17)	O1—C7—H7A	109.5
C1—C2—S1	121.8 (2)	O1—C7—H7B	109.5
C2—C3—C4	112.2 (2)	H7A—C7—H7B	109.5
C2—C3—H3	123.9	O1—C7—H7C	109.5
C4—C3—H3	123.9	H7A—C7—H7C	109.5
C5—C4—C3	112.0 (2)	H7B—C7—H7C	109.5
C5—C4—H4A	124.0		
			
C7—O1—C1—O2	3.1 (4)	C2—C3—C4—C5	−1.0 (3)
C7—O1—C1—C2	−176.3 (2)	C3—C4—C5—C6	−177.8 (2)
O2—C1—C2—C3	−6.1 (4)	C3—C4—C5—S1	0.8 (3)
O1—C1—C2—C3	173.3 (2)	C2—S1—C5—C4	−0.3 (2)
O2—C1—C2—S1	175.6 (2)	C2—S1—C5—C6	178.4 (2)
O1—C1—C2—S1	−5.0 (3)	C4—C5—C6—O3	175.4 (3)
C5—S1—C2—C3	−0.3 (2)	S1—C5—C6—O3	−3.1 (3)
C5—S1—C2—C1	178.2 (2)	C4—C5—C6—O4	−3.6 (4)
C1—C2—C3—C4	−177.6 (2)	S1—C5—C6—O4	177.90 (19)
S1—C2—C3—C4	0.8 (3)		

### Hydrogen-bond geometry (Å, °)

**Table:**

*D*—H···*A*	*D*—H	H···*A*	*D*···*A*	*D*—H···*A*
O4—H4···O3^i^	0.82	1.82	2.639 (2)	173

Symmetry codes: (i) −*x*, −*y*+1, −*z*+1.

## References

[bb1] Sheldrick, G. M. (1996). *SADABS* University of Göttingen, Germany.

[bb2] Sheldrick, G. M. (2008). *Acta Cryst.* A**64**, 112–122. 10.1107/S0108767307043930 18156677

[bb3] Siemens (1996). *SMART* and *SAINT* Siemens Analytical X-ray Instruments Inc., Madison, Wisconsin, USA.

[bb4] Zhao, L., Liang, J., Yue, G., Deng, X. & He, Y. (2009). *Acta Cryst.* E**65**, m722. 10.1107/S1600536809020273 PMC2969381 21582666

